# Abrasive Wear Resistance of Ultrafine Ausferritic Ductile Iron Intended for the Manufacture of Gears for Mining Machinery

**DOI:** 10.3390/ma16124311

**Published:** 2023-06-11

**Authors:** Dawid Myszka, Andrzej N. Wieczorek, Emilia Skołek, Tomasz Borowski, Krzysztof Kotwica, Marek Kalita, Radosław Kruk, Paweł M. Nuckowski

**Affiliations:** 1 Faculty of Mechanical and Industrial Engineering, Warsaw University of Technology, Narbutta 85 Street, 02-524 Warsaw, Poland; dawid.myszka@pw.edu.pl; 2 Faculty of Mining, Safety Engineering and Industrial Automation, Silesian University of Technology, Akademicka 2 Street, 44-100 Gliwice, Poland; 3 Faculty of Materials Engineering, Warsaw University of Technology, Wołoska 147 Street, 02-507 Warsaw, Poland; emilia.skolek@pw.edu.pl (E.S.); tomasz.borowski@pw.edu.pl (T.B.); 4 Faculty of Mechanical Engineering and Robotics, AGH University of Science and Technology, 30-059 Cracow, Poland; kotwica@agh.edu.pl; 5 Division of Machines and Equipment, KOMAG Institute of Mining Technology, Pszczyńska 37 Street, 44-101 Gliwice, Poland; mkalita@komag.eu; 6 Institute of Mechanical Engineering, Clausthal University of Technology, Robert-Koch-Straße 32, D-38678 Clausthal-Zellerfeld, Germany; kruk@imw.tu-clausthal.de; 7 Materials Research Laboratory, Faculty of Mechanical Engineering, Silesian University of Technology, Konarskiego 18A Street, 44-100 Gliwice, Poland; pawel.nuckowski@polsl.pl

**Keywords:** gears, abrasion wear, ausferritic ductile iron, austempering temperature

## Abstract

The purpose of this study was to experimentally determine the abrasion wear properties of ausferritic ductile iron austempered at 250 °C in order to obtain cast iron of class EN-GJS-1400-1. It has been found that such a cast iron grade makes it possible to create structures for material conveyors used for short-distance transport purposes, required to perform in terms of abrasion resistance under extreme conditions. The wear tests addressed in the paper were conducted at a ring-on-ring type of test rig. The test samples were examined under the conditions of slide mating, where the main destructive process was surface microcutting via loose corundum grains. The mass loss of the examined samples was measured as a parameter characteristic of the wear. The volume loss values thus obtained were plotted as a function of initial hardness. Based on these results, it has been found that prolonged heat treatment (of more than 6 h) causes only an insignificant increase in the resistance to abrasive wear.

## 1. Introduction

The current market of technical machinery and equipment gives particular preference to products characterised by relatively low production costs, yet offering high mechanical strength. One of the possible applications of the available manufacturing methods can be, for example, the production of cost-effective gears for industrial transmissions. The latter are products of complex geometry which require a long service life, while the cost of their production is relatively high. The global market of power transmission elements, including gears and chain wheels [1], is huge and a significant amount of these products are also manufactured using casting methods on account of the low production costs involved; however, these gears are characterised by low strength parameters.

With regard to the category of low power transmission gears (e.g., up to 30 kW), using advanced gear production technologies, including processes such as forging, machining, grinding, and surface heat treatment, significantly increases their costs. The production costs are expected to be reduced when shafts, pinions, and gears with pre-grooved teeth are cast, which enables significant savings in material, labour, tooling, and energy costs.

The production of gears using casting methods is not an easy technological process when considering the need to ensure that the elements being manufactured display sufficient resistance to certain operational factors [2,3,4,5] such as the abrasive impact of oil contaminant particles and variable dynamic forces [6,7,8,9]. The gears for mining applications, used in lightweight conveyors, are exposed to variable external loads and internal excitation caused by, among other factors, stiffness fluctuations and manufacturing errors [10,11]. This significantly affects the resistance of the teeth to fatigue fracture at the root. Growing load variability causes the dynamic load component to increase, which, in turn, negatively affects the durability of gears.

**Figure 1:**
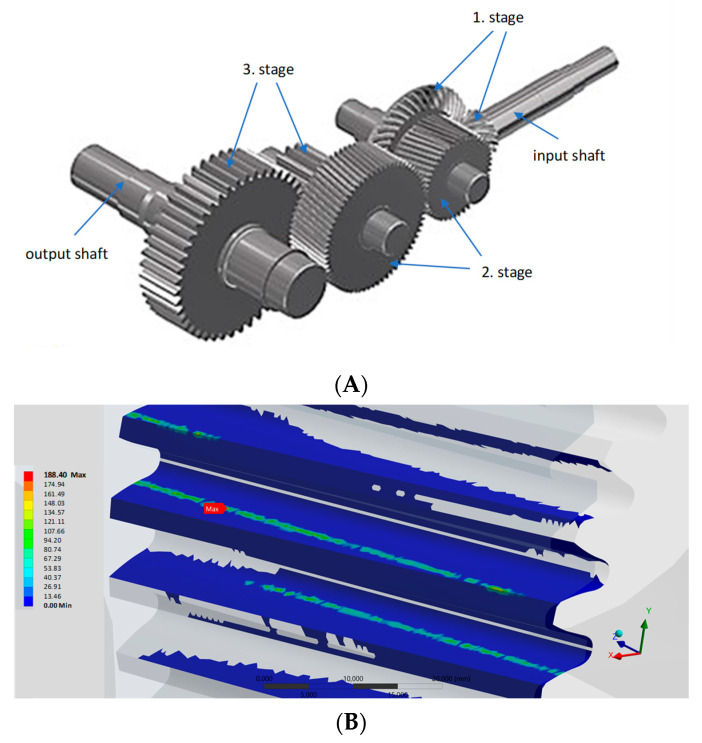

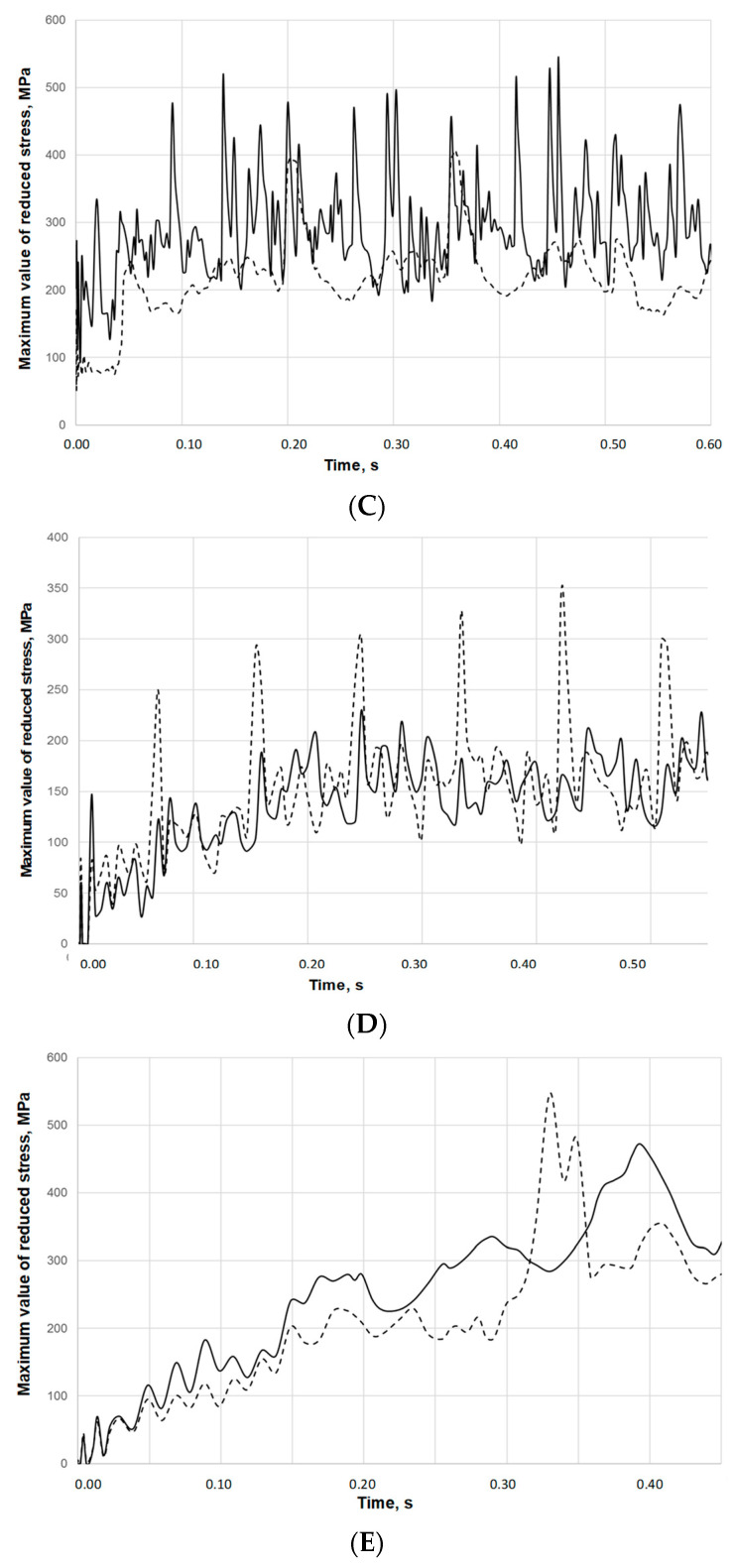
Demonstration load analysis of mining gear teeth; (**A**)—transmission model, (**B**)—contact stresses determined for the second gear stage (input rotational speed *n* = 448.64 RPM, torque T = 472.75 Nm and gear ratio i = 1.51), (**C**)—curves of the maximum reduced stress value determined for the first gear stage; (**D**)—curves of the maximum reduced stress value determined for the second gear stage; (**E**)—curves of the maximum reduced stress value determined for the third gear stage; results determined for the pinion gear are presented with a dashed line, and with a solid line for the gear wheel.

Variable dynamic forces also have a negative impact on the resistance of these gears to fatigue surface chipping [12]. Figure 1 illustrates the results of a simulation of the contact stresses in a low power transmission used in the mining industry. Calculations were made using the Ansys software for the following data: input rotational speed *n* = 1470 RPM, torque T = 191 Nm. It clearly shows the large variability of the pressure values in the contact area of individual teeth.

**Figure 2:**
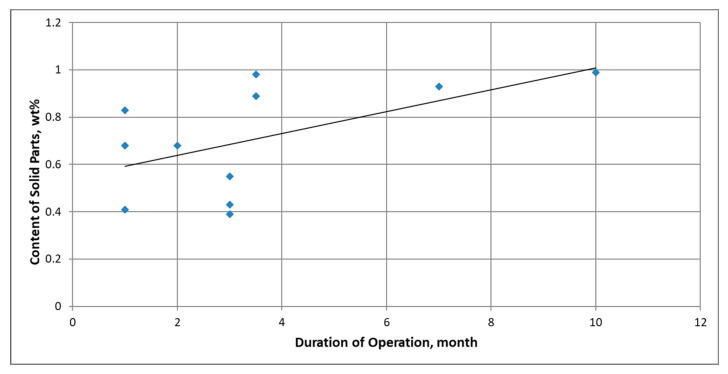
Effect of operation time on the content of solids parts (wt%) in the oil used to lubricate mining transmission gears.

Figure 2 shows the impact of the operation time of mining gears on the content of solid impurities in lubricating oils. It is evident that for an average oil change interval of 1 year, the mass fraction of these impurities exceeds 1%, which significantly exceeds the thresholds acceptable by the manufacturers of these machines and suppliers of oils (permissible level of solid impurities is 0.02% [2]). This implies the need to use materials resistant to abrasive wear.

The casting materials used to date, such as grey cast iron and cast alloy steel, are not intended for operation under mining conditions due to their susceptibility to cracking and relatively low impact strength [13]. Casting materials that meet these requirements to some extent are ausferritic ductile irons (ADI).

**Figure 3:**
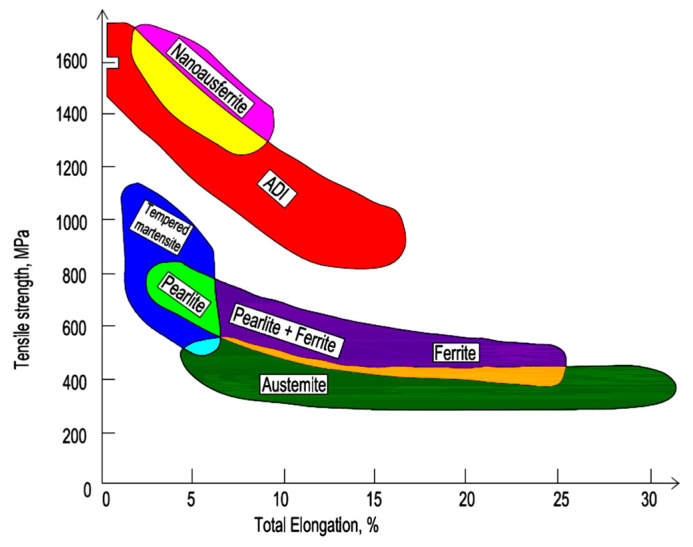
Mechanical properties of ductile iron with various types of matrix; based on [13].

Austempered ductile iron (ADI) is a material made from ductile iron following special heat treatment by means of isothermal quenching [13,14]. The starting nodular iron for ADI production most often contains Mo, Ni and Cu, which increases the material’s hardenability and ductility. Ductile iron based on an ausferritic matrix is a popular and highly valued structural material [15,16,17] (see Figure 3). The above remark results from the fact that ADI cast irons are a material that provides relatively high mechanical properties with high wear resistance resulting from the TRIP effect under load. Similarly to high-strength steel with a fine bainite structure, this grade of cast iron can be obtained by austempering at low temperatures of several dozen degrees more than the MS temperature [18,19].

Compared to steel, cast iron offers certain advantages, such as density being only 10% lower, reduced manufacturing costs, and superior damping capacity. However, it also has some disadvantages, requiring engineers to handle this material with a high level of knowledge and skill. Additionally, it is commonly known that the more homogenous and refined the structure, the better the properties of cast iron. However, the main problem is the microstructural heterogeneity which results from the specific production regime of castings and the structure which develops during solidification. Due to this heterogeneity, the isothermal transformation forms structures with varied morphologies in the cast iron matrix.

As aforementioned, ADI’s structure is composed of ferrite plates in austenite and areas of block austenite characterised by considerable metastability. Such a combination of phases makes ADI resistant to tribological wear [20,21] and cracking. First of all, austempered ductile iron shows a tendency towards surface hardening as a result of the phase transformation of austenite [22,23,24,25,26] into martensite under load. This effect is attributable to the fact that austenite subjected to deformation [27,28,29,30,31,32,33,34], meeting specific conditions, is transformed into martensite, referred to as deformation martensite.

Depending on the heat treatment procedure performed, ADI cast irons differ in terms of phase fractions, which significantly affects their impact strength and resistance to brittle cracking [35,36,37]. It has been found that a material with a microstructure consisting of packets of thin ferrite plates separated by stable austenite will require more energy to tear it off from the substrate during abrasion [38,39,40]. A material characterised by higher plastic properties and a higher content of austenite will behave similarly in strain hardening.

On account of their known resistance to abrasive wear, ADI cast irons are already used in open gear transmissions of mining machines, such as ball mills. However, they have not yet been used in the construction of gears for the drive systems of transport machines due to the increased dynamic impact accompanying their operation [41,42,43]. In order to improve the dynamic load resistance, the authors proposed a new material—ultrafine ausferritic ductile iron.

A review of the literature on the subject implies that the size of lamellar ferrite precipitates is the main factor determining the value of elastic strain in ausferritic ductile iron. This means that the finer the ferrite in the cast iron matrix, the better the strength properties. There are many studies on the effects attributable to the ultra-fineness of phase components on the functional properties of various materials (e.g., [44,45,46,47,48,49,50,51]); however, relatively few papers address ADI cast irons only.

The effect obtained by Azevedo et al. [52] in the process of rapid austenitisation of former martensitic structures was the fragmentation of austenite grains. Panneerselvam [53] studied the impact of factors such as cryogenic treatment, intercritical austenitisation with one- and two-stage quenching, and high-temperature plastic deformation on the microstructure and mechanical properties of ductile low-alloy cast iron. Myszka et al. [54,55] demonstrated that a longer time of isothermal transformation in the lower range of ausferritic transformation temperatures can result in significant fragmentation of the ferrite plates with a grain size below 100 nm.

**Figure 4:**
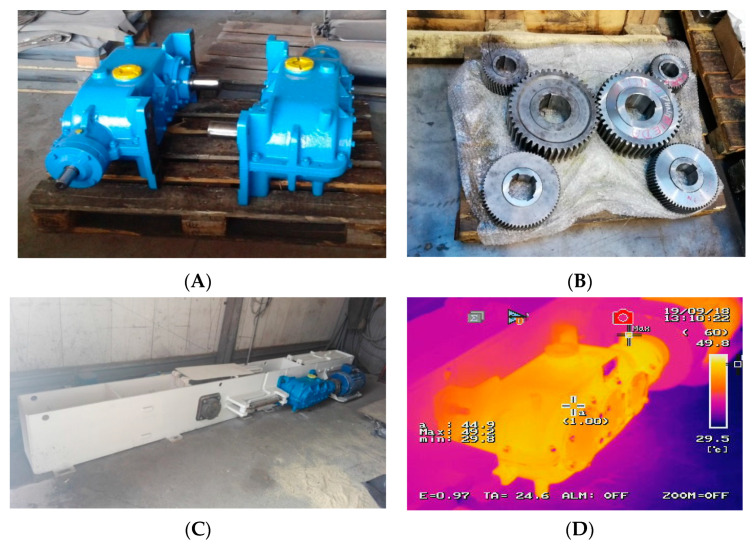
Prototype transmission featuring gears made of ultrafine ausferritic ductile iron; (**A**)—prototype transmissions, (**B**)—gears made of ultrafine ausferritic ductile iron, (**C**)—transmission at a test rig, (**D**)—sample transmission thermogram obtained during the tests.

With reference to the aforementioned papers, the authors of this study developed a technology for the manufacture of industrial gears from ADI with ultra-fine structure, and further produced a prototype gear for mining conveyor drives (Figure 4A,B). The prototype transmission has been designed for the ratios of 1:13, 1:19 and 1:29 as an enclosed unit, capable of operating in both directions, and mineral oil-bath lubricated. The variant tested for durability was the 1:13 transmission. The tests conducted under operating conditions confirmed the high-strength properties of this type of structure (Figure 4C,D).

This study follows the research on the practical implementation of a new technology of heat treatment in terms of the understanding of the anti-wear properties of the ultrafine ausferritic structure. The aim of this research was to determine the effect of morphology and phase fractions on the abrasive wear of ADI with very fine grain structure. The study represents a part of a more comprehensive research aimed at determining the effect of a nanometric phase size on the properties of castings.

For comparative purposes, two types of steels were taken into account, both typically used in the manufacture of the toothed elements of mining gear transmissions for lightweight under-wall scraper conveyors. Given the current state of knowledge, the novel aspect of this study is the determination of the susceptibility of ultrafine ausferritic ductile iron to abrasive wear against the steel grades previously in use.

## 2. Experimental Details

### 2.1. Characteristics of the Testing Methodology

**Figure 5:**
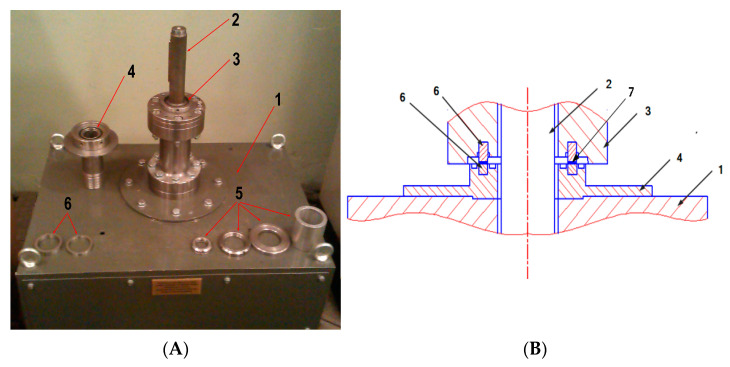
The design of the test rig; (**A**)—view of the test rig, (**B**)—diagram showing the manner of fixing the samples: designations: 1—body of the test rig with a motor, 2—drive shaft, 3—upper sample holder, 4—lower sample holder, 5—fastening elements, 6—test samples, 7—abrasive [56].

Abrasive wear tests (Figure 5) were performed at a rig designed and built at the Institute of Mining Mechanisation (the test itself has been described in more detail in other studies [56]). The main friction pair consisted of two ring-shaped samples, between which some abrasive material was inserted. During the tests, corundum grains with a diameter below 50 μm were used as the abrasive material. They were placed between the mating samples in an amount of 1 cm^3^.

Measurements were taken under four different variants of the compressing stress applied to the samples, i.e., at 0.031, 0.062, 0.094, and 0.125 MPa. The research methodology assumed that following each 10 min wear cycle, the samples would be cleaned and weighed, and some fresh corundum abrasive would be added.

The basic parameters characterising the wear test have been provided in Table 1. The wear parameter to be determined was defined according to Formula (1):

(1) $$V_{W}=\frac{u_{M}}{\rho }=\frac{\left(m_{pd0}-m_{pdt}\right)+\left(m_{pg0}-m_{pgt}\right)}{\rho }$$

where *V_w_*—volumetric wear; mm^3^; *u_M_*—loss of the sample mass; g; *m_pd_*_0_—mass of the bottom sample before the test; g; *m_pdt_*—end mass of the bottom sample; g; *m_pg_*_0_—mass of the top sample before the test; g; *m_pgt_*—end mass of the top sample; g; *ρ*—mass density, g/mm^3^.

The uncertainty of the mass wear was specified for the confidence level of 0.95 and the number of repetitions of *n* = 3. The relative measurement uncertainty was less than 3% for all the cases considered. The samples subjected to abrasion were cut and a series of tests was conducted in their cross-sections, including microscopic examinations via light microscopy as well as scanning electron microscope measurements. The microscopic wear examinations were conducted at magnifications of up to ×1000 by identifying the edge working under the conditions of abrasive wear and showing the characteristics of abraded surface at magnifications of ×300.

### 2.2. Characteristics of the Materials Tested

The material chosen for the tests based on previous studies [53,54] was characterised by a chemical composition comprising 1.9% Ni and 0.9% Cu. The cast iron was produced by remelting pig iron, steel, ferroalloys, and alloying additives. The metallurgical process was performed in the PI35 medium frequency induction furnace with a capacity of 40 kg. Spheroidisation was conducted via the sandwich method using FeSiMg added to the pouring ladle. The casting samples, with the chemical composition provided in Table 2, were poured into mould cavities of the YII geometry, made of core mass. In order to obtain ausferritic ductile iron, the casting was followed by heat treatment procedures, including austenitisation in a furnace (Nabertherm, Lilienthal, Germany) and oil austempering at a temperature of 250 °C (Table 3). The chemical composition and the heat treatment conditions of the reference steels are also provided in Table 2 and Table 3.

**Figure 6:**
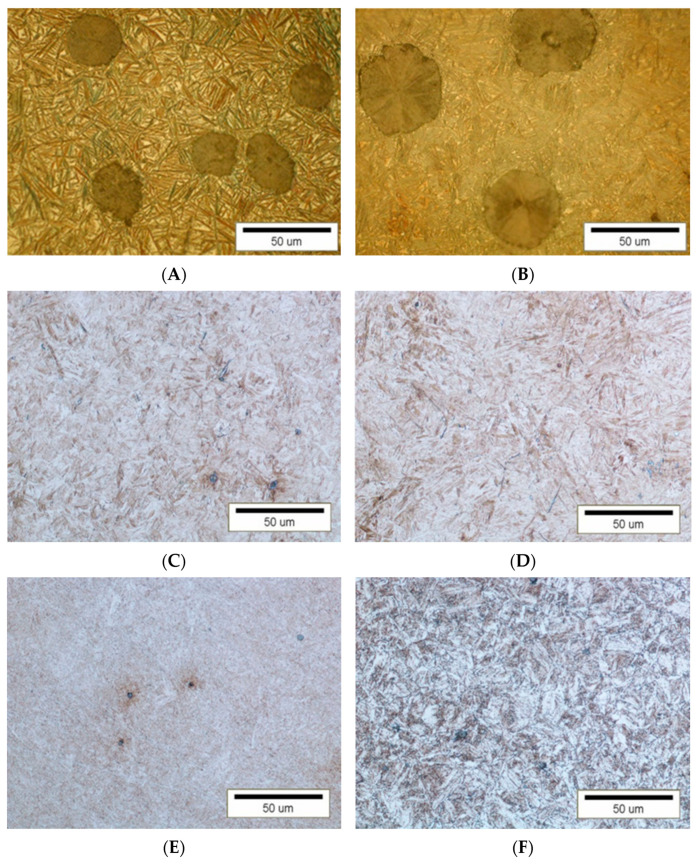
Microstructure of the cast irons and steels tested, (**A**)—ADI 250_6h, (**B**)—ADI 250_24h, (**C**)—34CrNiMo-6 (H) (×500), (**D**)—30CrMo-12 (H) (×500), (**E**)—34CrNiMo-6 (Q + T) (×500), (**F**)—30CrMo12 (Q + T) (×500); etched state (5% Nital).

**Figure 7:**
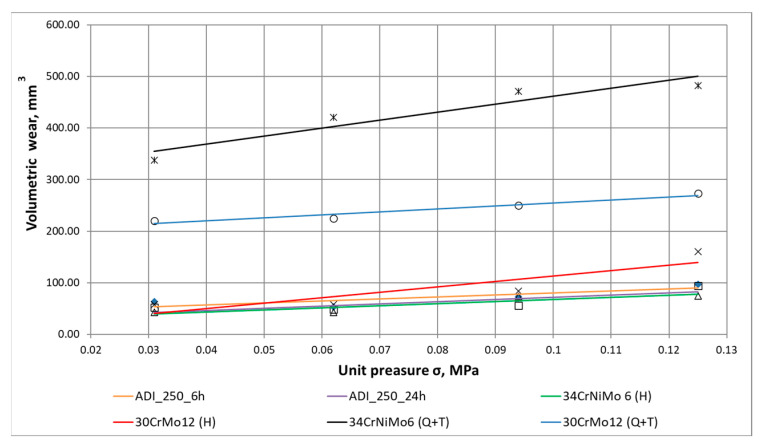
Graphs of volumetric wear of the materials tested in a function of compressive stress.

**Figure 8:**
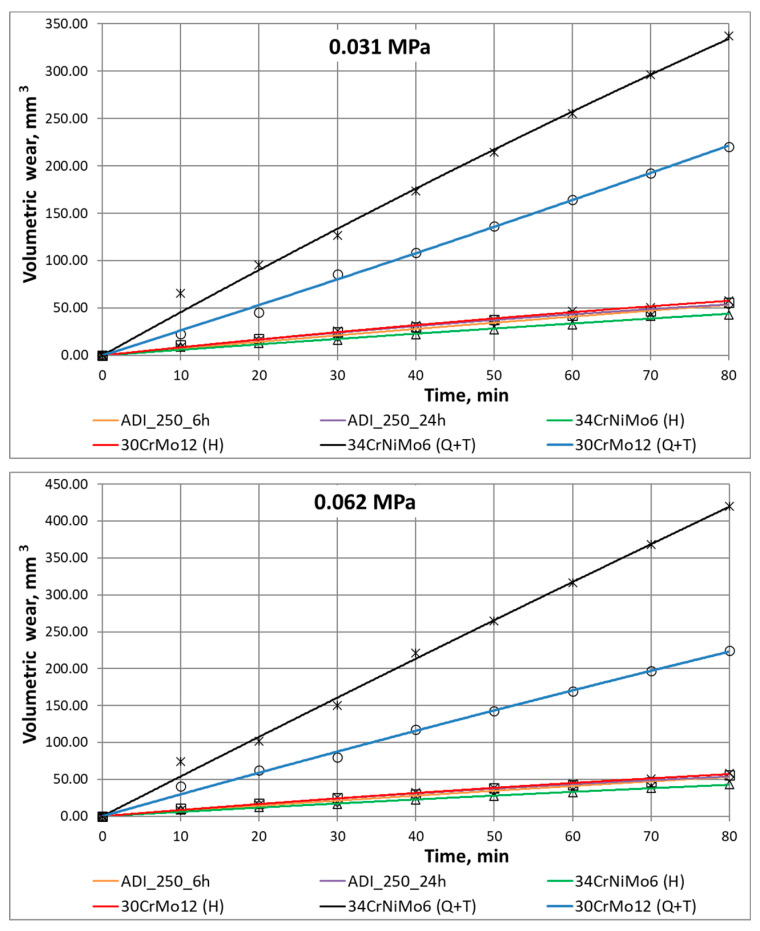

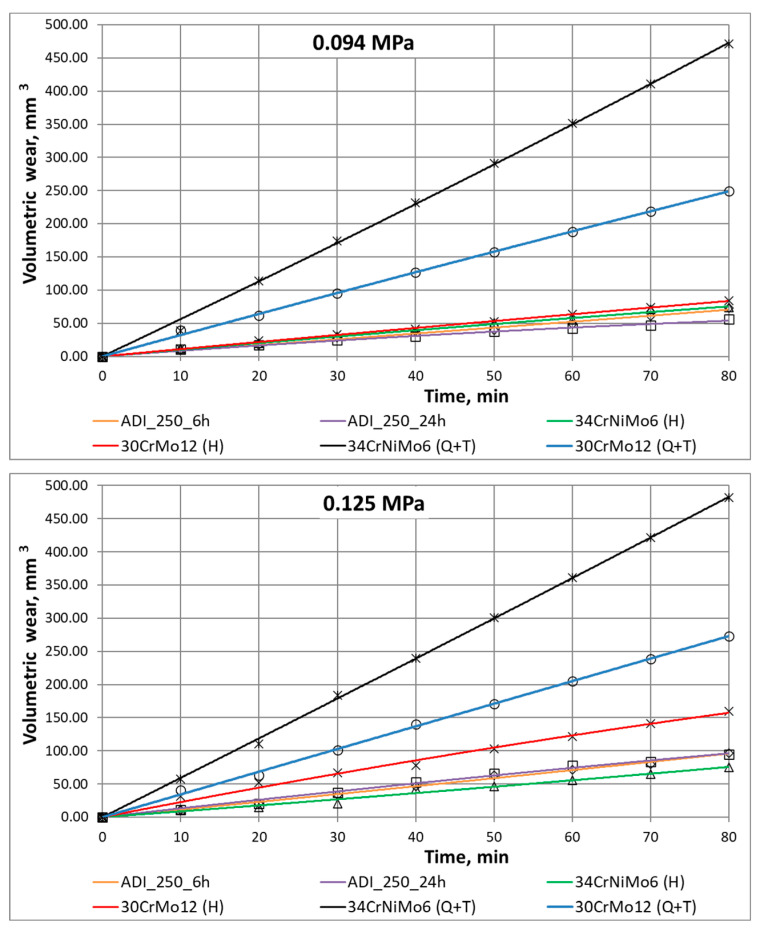
Graphs of volumetric wear of the materials tested in a function of wear time.

Next, samples were cut for purposes of hardness and abrasive wear test. Their microstructures are shown in Figure 6, Figure 7 and Figure 8. The microstructure assessment was performed through image analysis in ImageJ using scanning electron (S-3500N, Hitachi, Tokyo, Japan) and optical (Eclipse LV-150, Nikon Metrology Inc., Tokyo, Japan) microscopy photos.

The matrix of austempered ductile irons (ADI) (Figure 6A,B) shows the lower ausferrite structure, this being more fine-grained in the ADI 250_24h variant. When case-hardened, the 34CrNiMo6 and 30CrMo-12 steels (Figure 6C,D) show a uniform structure of tempered martensite with small amounts of retained austenite, while the structure of both steels following quenching and tempering is sorbitic (Figure 6E,F).

Table 4 provides the hardness of the austempered ductile iron and of the 34CrNiMo-6 and 30CrMo-12 steels after hardening as well as quenching and tempering. The test material was prepared so that the fragmentation of the resulting ferrite plates was similar; however, the materials differed in terms of the austenite content. It should be noted that blocky austenite is responsible for deformation-induced martensitic transformation. After a longer time of transformation, the austenite content decreases, and the ferrite content increases, as shown in Table 5. This is one of the reasons why the hardness of ductile iron is lower compared to that of steels.

The X-ray diffraction analysis was carried out on an Panalytical X’Pert Pro diffractometer (Panalytical, Almelo, The Netherlands), with the use of X-ray lamp radiation with a cobalt anode (KαCo λ = 1.7909 Å), powered by voltage 40 kV, with the filament current intensity = 30 mA. The X-ray diffraction measurements were performed in the Bragg–Brentano geometry in the angular scope 30–120° [2θ] with the step 0.05° and the step count time 100 s.

The obtained diffractograms were analysed using X’Pert High Score software v. 3.0e with a dedicated Inorganic Crystal Structure Database—ICSD (FIZ, Karlsruhe, Germany) The quantitative share of crystalline phases was carried out using the Rietveld method. X-ray phase identification tests were performed for samples in the initial state and after wear tests. The diffractograms obtained for the cast iron variant ADI 250_6h are presented in Chapter 3.

## 3. Results and Discussion

The way in which the abrasive medium acts on a sample fixed at the test rig for wear resistance studies simulates the actual operation of mining equipment components. The tests, whose results have been depicted in Figure 7 and Figure 8, show that in the case of low and medium unit pressure (0.031–0.094 MPa) applied at the test rig, the lower wear rate corresponds to the cast iron austempered for 24 h compared to that austempered for 6 h.

The highest load yields a wear rate similar for both variants. This means that for the extreme conditions of abrasion, the defence mechanisms protecting the material against wear failure and chipping due to single abrasive grains take action in areas characterised by strong deformation. Hardened steels show a low wear rate similar to austempered cast irons with a slightly higher value of wear under the highest load of 0.125 MPa for the 34CrNiMo-6 steel. Steels following quenching and tempering demonstrate rates several times higher. Having analysed the results obtained for the highest load (0.125 MPa), one can notice that the 34CrNiMo-6 steel shows the best abrasive wear resistance after the hardening process, in which it performs better than cast iron; however, in turn, the 30CrMo-12 steel shows a higher wear rate, which can be caused by the differences in the toughness of these materials. Quenched and tempered steels are characterised by the lowest hardness, which manifests itself in the highest wear volumes.

The analysis of the abrasion process in the cases of the two extreme loads has yielded very interesting results. Figure 8 shows that in the first stage of the short-time abrasion test, both materials behave in a similar manner. With a longer time of abrasion, the material containing more austenite blocks becomes less resistant to wear. In the case of high loads, it becomes evident that the time of abrasion is no longer decisive of the nature of wear, and both of the materials tested show similar wear behaviour. The values obtained in a function of time imply that the wear kinetics represents a typical process featuring both a starting phase and a stabilised wear phase.

**Figure 9:**
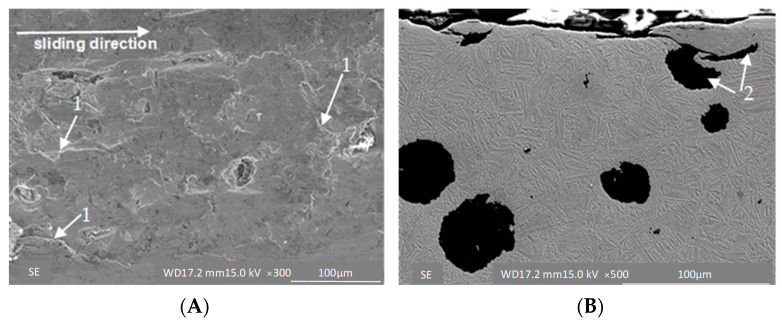
Surface morphology (**A**) and microstructure (**B**) of the ADI 250_6h samples exposed to abrasion under unit pressure of σ = 0.031 MPa; 1—cracks, 2—deformed graphite.

In the case of the ADI 250_6h sample, small cracks are evident, which suggests chipping of the fine fragments of material on the surface (Figure 9A). This is clearly visible in the cross-section of the abraded surface (Figure 9B), where the cracks are attributable to the presence of graphite in the structure and to the strong plastic deformation resulting in the occurrence of hard martensite fields.

**Figure 10:**
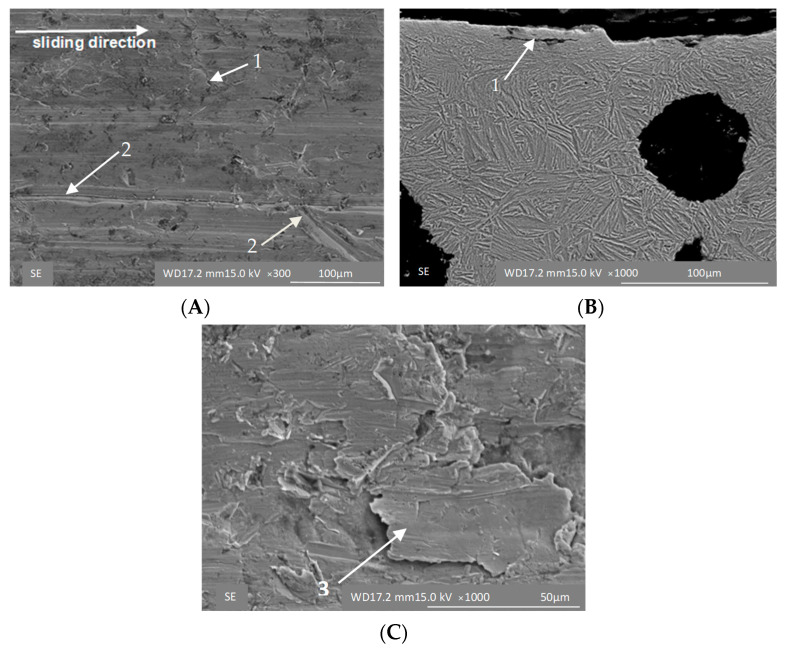
Surface morphology (**A**,**C**) and microstructure (**B**) of the ADI 250_24h samples exposed to abrasion under unit pressure of σ = 0.031 MPa; 1—cracks, 2—scratch, 3—delamination.

**Figure 11:**
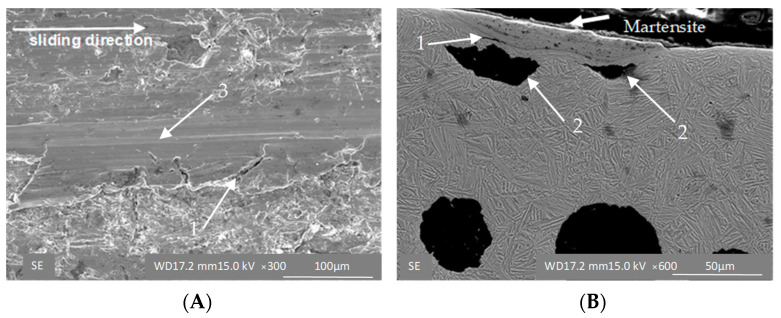
Surface morphology (**A**) and microstructure (**B**) of the ADI 250_6h samples exposed to abrasion under unit pressure of σ = 0.125 MPa; 1—cracks, 2—deformed graphite, 3—scratch.

**Figure 12:**
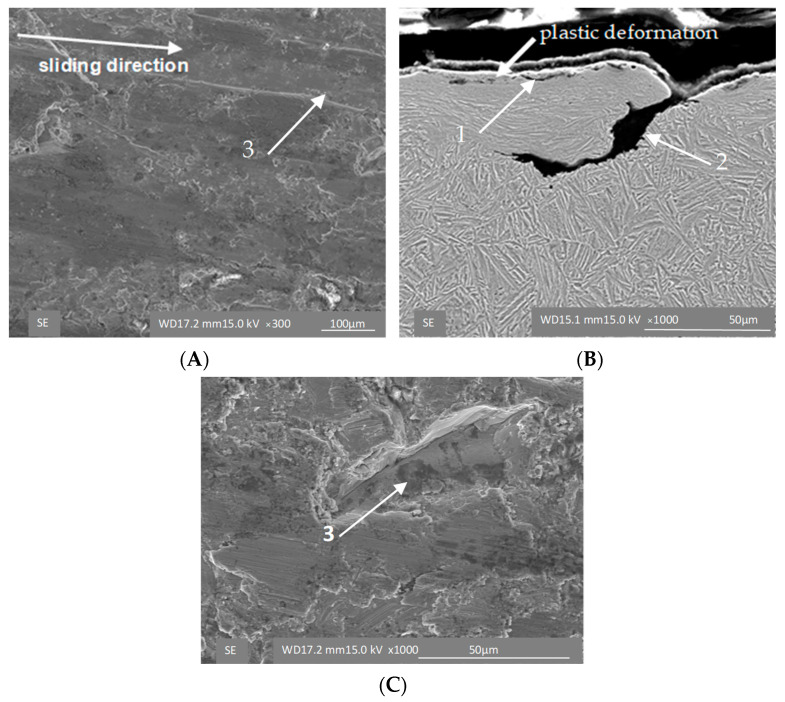
Surface morphology (**A**,**C**) and microstructure (**B**) of the ADI 250_24h samples exposed to abrasion under unit pressure of σ = 0.125 MPa; 1—cracks, 2—deformed graphite, 3—delamination.

The strain-induced transformation manifests itself particularly clearly under high loads. The surface of the ADI 250_24h sample shows signs of scratches characteristic of the cast iron with lower austenite content (Figure 10A) and small cracks in the cross-section (Figure 10B). Under the high load (σ = 0.125 MPa), which simulates extremely harsh wear conditions, different mechanisms are in action in the two materials tested. In the case of the ADI_250_6h sample, the microstructure is composed of martensite, which is responsible for the brittle fracture of the material (Figure 11), while the ADI 250_24h sample shows the strain-controlled mechanism of the material hardening (Figure 12).

It manifests itself as the shallow scratches of the abraded surface (Figure 11A and Figure 12A) and as the deformation of the ausferritic structure in the superficial layer of the cast iron (Figure 11B and Figure 12B). In addition to the types of damage mentioned above, delamination of the surface layer was also observed (Figure 10C and Figure 12A,C). As shown in Figure 9, in the initial phase of the test, the surface of the samples was exposed to very strong impacts of fresh sharp grains. This can activate more effective material removal from the surface and graphite deformation. On the other hand, it may result in surface hardening by way of the TRIP (transformation induced plasticity) effect (Figure 11B) of the blocky austenite in micro-areas and detachment of superficial pieces of samples.

In the subsequent stages of the tests, the abrasive grains were broken, the material was removed from the mating surfaces and completed with the remaining fraction of heavily crushed abrasive causing strong surface deformations (Figure 9B and Figure 12B). The superfinishing phase was the cause of the slight differentiation in the surface image of the cooperating samples visible under the SEM microscope.

The abrasive wear of cast iron is primarily affected by the conditions in which the wear processes take place; however, the structure of cast iron and the properties of its components have a significant impact on the intensity of wear. Abrasive wear is usually directly related to the material hardness and depends on the ratio between the hardness of the alloy and the hardness of the abrasive [57]. Table 6 presents the average hardness of the component phases of ADI cast irons. It is easy to see, for austenite and pearlite, an increase in their hardness in the case of alloy cast irons [13]. Therefore, in order to improve the abrasion resistance of cast iron, alloying elements forming hard phases are added [13,25,26,35].

In the case of cast iron with ausferritic matrix, no clear relationship between hardness and material wear was established. It appears that the slightly lower hardness of ausferritic cast iron does not make it uncompetitive with a steel grade of 600 HV in hardness. This is affected by the aforementioned surface-hardening ability of ADI cast iron through mechanical action until critical stresses are exceeded, causing martensitic deformation and the effect of strain-hardening by twinning. The surface hardening and the special microstructure of ADI cast iron are both reflected in the increased wear resistance.

**Figure 13:**
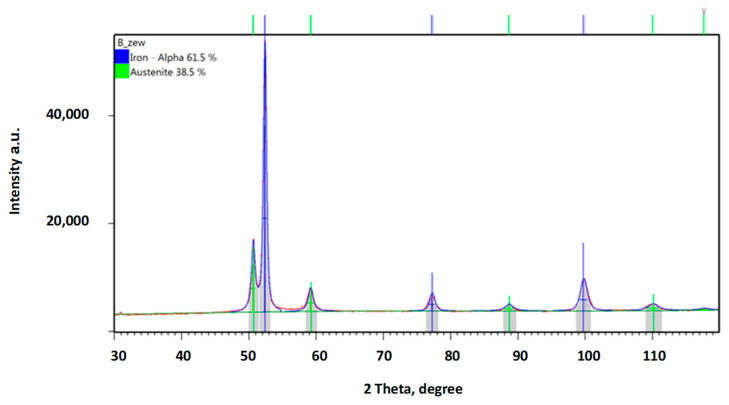
Diffraction patterns obtained for ADI 250_6h; measurement in an unloaded area.

**Figure 14:**
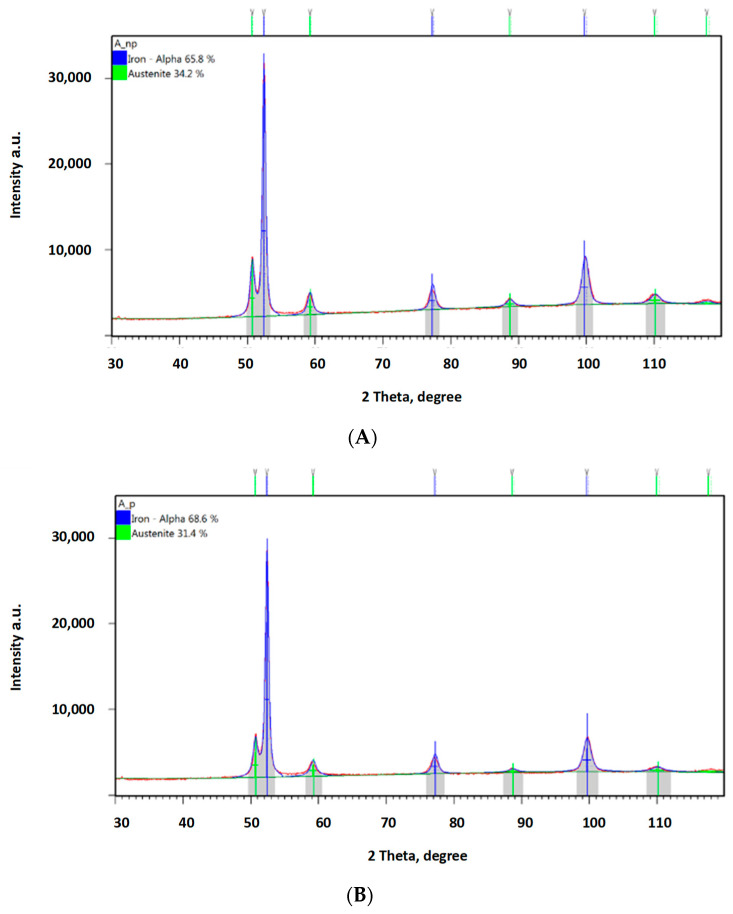
Diffraction patterns obtained for ADI 250_6h; (**A**)—measurement in an area subject to a load of 0.031 MPa without abrasive effect, (**B**)—measurement in an area subject to a load of 0.031 MPa with abrasive effect.

**Figure 15:**
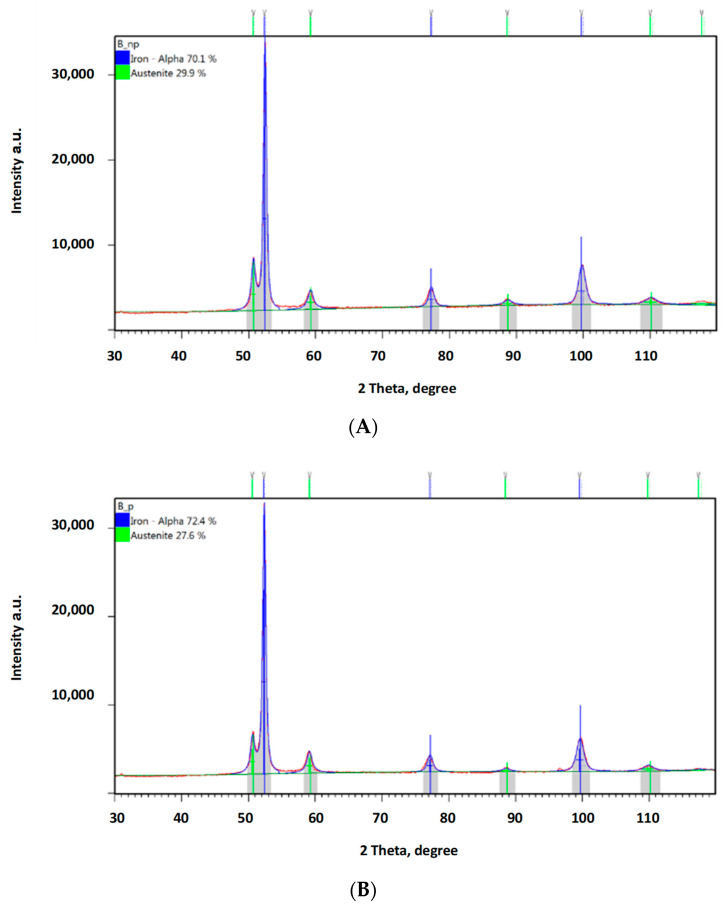
Diffraction patterns obtained for ADI 250_6h; (**A**)—measurement in an area subject to a load of 0.062 MPa without abrasive effect, (**B**)—measurement in an area subject to a load of 0.062 MPa with abrasive effect.

**Figure 16:**
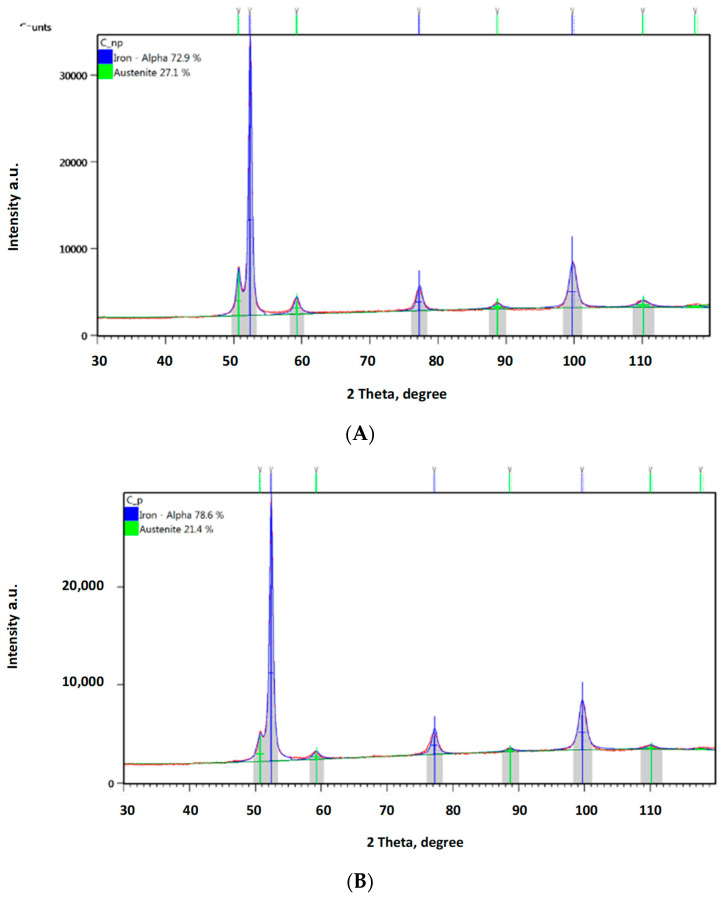
Diffraction patterns obtained for ADI 250_6h; (**A**)—measurement in an area subject to a load of 0.094 MPa without abrasive effect, (**B**)—measurement in an area subject to a load of 0.094 MPa with abrasive effect.

In order to confirm the foregoing, additional phase composition tests were performed using the XRD method. Figure 13 provides a diffraction pattern obtained from a sample not subject to any loading. The austenite content in this sample was ca. 38%, while all of the remaining structure was ferrite. The hardening effect was observed not only in the abrasive impact zone (Figure 14A, Figure 15A and Figure 16A), but also at the bottom of the specimen, which was caused by the pressure itself (Figure 14B, Figure 15B and Figure 16A).

Both groups of results clearly show a declining austenite content due to the effect of the load applied. Analysis of the zones subject to the impact of abrasive grain revealed the following reductions in the austenite content compared to the reference value: by ca. 4.3% for a load of 0.031 MPa; by ca. 8.6% for a load of 0.062 MPa; and by ca. 11.4% for a load of 0.094 MPa. The austenite content reduction values were higher where the surface was subject to wear in the presence of abrasive grain: by ca. 7.1% for a load of 0.031 MPa; by ca. 10.9% for a load of 0.062 MPa; and by ca. 17.1% for a load of 0.094 MPa. What explains the higher austenite content reduction values are the higher values of local pressure attributable to the impact exerted via a smaller area of individual grains.

The reduced share of austenite in the structure proves that it was subject to a transformation-induced plasticity (TRIP) effect, causing the martensite content to increase in the contact zone. The diffraction patterns show that the ferrite and martensite peaks did not coincide; however, the fact that the austenite content declined suffices to confirm the case-hardening effect.

Having analysed the wear test results obtained, one can clearly notice that the quenched and tempered steels were characterised by the lowest abrasive wear resistance, while the case-hardened steels and the ADI of ultra-fine microstructure displayed comparable volumetric wear parameters. This provides the rationale for recommending these cast irons as an alternative material for the production of gear wheels for light-duty transmissions, making it possible to reduce production costs.

## 4. Conclusions

The multitude of cast iron types arising from the use of various heat treatment parameters and leading to diverse effects, including the formation of nanostructured grain, offer an extremely wide range of properties.

In respect to the subject of the study, concerning the potential applications of cast irons with ultrafine ausferritic ductile iron, it should be noted that the test results discussed in thid paper imply that these materials can be used in the production of gears on account of their tribological properties.

With reference to these test results, the following specific conclusions can be formulated:

1. Ultrafine ausferritic ductile iron exhibits comparable wear resistance to hardened alloy steels.
2. Under the influence of abrasive grains, a transformation from austenite to martensite occurs in ultrafine ausferritic ductile iron.
3. The effect of surface strengthening in ADI cast irons also occurs under the influence of pressure alone, but it is smaller compared to the effect of the abrasive.
4. Micro-scratching, cracking and delamination are typical of abrasive damage in ultrafine ausferritic ductile irons.
5. Under the influence of friction, deformation of graphite spheres is observed, which may favour the formation of surface cracks.

## Figures and Tables

**Table 1:** Main parameters of the wear tests.

Parameter	Value			
Contact surface area S, mm^2^	785.3			
Compressing stress σ, MPa	0.031	0.062	0.094	0.125
Tests duration, min	8 × 10	8 × 10	8 × 10	8 × 10
Sliding distance, m	1390	1390	1390	1390
Mass of the set load, kg	2.5	5.0	7.5	10.0
Rotational speed of the moving sample, RPM	149.1			
Average linear speed of the moving sample, m/s	0.29			
Number of test repetitions for each variant	3			
Outside diameter of the sample	Ø55h8			
Inside diameter of the sample	Ø45H7			
Sample width (B)	for upper lid B = 10 mm, for lower lid B = 6 mm			

**Table 2:** Chemical composition of ductile iron and steels [mass%].

Material	C	Si	Mn	Cu	Ni	Mg	Cr	Mo
ADI	3.60	2.45	0.32	0.93	1.90	0.065	0.002	0.08
34CrNiMo-6	0.33	0.31	0.60	0.18	1.39	0.003	1.33	0.02
30CrMo-12	0.31	1.36	0.85	0.12	0.11	0.002	1.19	0.17

**Table 3:** Heat treatment parameters applied to the ductile iron and steels tested.

**Sample Designation**	**Heat Treatment**			
	**Austenitising**		**Austempering**	
	**°C**	**h**	**°C**	**h**
ADI 250_6h (austempered) *	900	0.5	250	6
ADI 250_24h (austempered) *			250	24
**Sample Designation**	**Austenitising**		**Austenitising**	
	**°C**	**°C**	**°C**	**h**
34CrNiMo-6 (Q + T)	860	2	600	3
34CrNiMo-6 (H)			-	-
30CrMo-12 (Q + T)			600	3
30CrMo-12 (H)			-	-

* process parameters were determined on the basis of work [40].

**Table 4:** Hardness of the materials analysed.

Sample Designation	Hardness, HRC	Sample Designation	Hardness, HRC
ADI 250_6h	37 ± 1	34CrNiMo-6 (Q + T)	34 ± 1
ADI 250_24h	39 ± 1	30CrMo-12 (H)	54 ± 2
34CrNiMo-6 (H)	56 ± 2	30CrMo-12 (Q + T)	29 ± 1

**Table 5:** Austenite and ferrite content in ADI cast iron.

Sample Designation	Austenite Blocks	Average Width of Austenite/Ferrite Plates
	µm^2^	nm
ADI 250_6h	5.1	77/175
ADI 250_24h	2.6	70/178

**Table 6:** Comparison of the hardness of the components of the cast iron structure [13].

Cast Iron Phases	Hardness HV	Cast Iron Phases	Hardness HV
Unalloyed ferrite	70–200	Ausferrite	280–550
Unalloyed austenite	170–230	Martensite	500–1010
Alloyed austenite	250–600	Cementite	840–1100
Unalloyed perlite	250–320	(Cr, Fe)_7_C_3_	1200–1600
Alloyed perlite	300–460	-	-

## Data Availability

Not applicable.
